# Design of a community-based intergenerational oral health study: “Baby Smiles”

**DOI:** 10.1186/1472-6831-13-38

**Published:** 2013-08-06

**Authors:** Peter Milgrom, Christine A Riedy, Philip Weinstein, Lloyd A Mancl, Gayle Garson, Colleen E Huebner, Darlene Smolen, Marilynn Sutherland

**Affiliations:** 1 Northwest Center to Reduce Oral Health Disparities, Department of Oral Health Sciences, University of Washington, Box 357475, 98195-7475 Seattle, WA, USA; 2 Northwest Center to Reduce Oral Health Disparities, Department of Health Services, University of Washington, Box 357230, 98195-7230 Seattle, WA, USA; 3 Klamath County Public Health, 403 Pine Street, Klamath Falls, OR, USA

**Keywords:** Counseling, Motivation, Dental caries, Early childhood caries, Health Education, Dental, Mothers, Infant

## Abstract

**Background:**

Rural, low-income pregnant women and their children are at high risk for poor oral health and have low utilization rates of dental care. The Baby Smiles study was designed to increase low-income pregnant women’s utilization of dental care, increase young children’s dental care utilization, and improve home oral health care practices.

**Methods/design:**

Baby Smiles was a five-year, four-site randomized intervention trial with a 2 × 2 factorial design. Four hundred participants were randomly assigned to one of four treatment arms in which they received either brief Motivational Interviewing (MI) or health education (HE) delivered during pregnancy and after the baby was born. In the prenatal study phase, the interventions were designed to encourage dental utilization during pregnancy. After childbirth, the focus was to utilize dental care for the infant by age one. The two primary outcome measures were dental utilization during pregnancy or up to two months postpartum for the mother, and preventive dental utilization by 18 months of age for the child. Medicaid claims data will be used to assess the primary outcomes. Questionnaires were administered at enrollment and 3, 9 and 18 months postpartum (study end) to assess mediating and moderating factors.

**Discussion:**

This trial can help define the most effective way to provide one-on-one counseling to pregnant women and new mothers regarding visits to the dentist during pregnancy and after the child is born. It supports previous work demonstrating the potential of reducing mother-to-child transmission of *Streptococcus mutans* and the initiation of dental caries prevention in early childhood.

**Trial registration:**

ClinicalTrials.gov Identifier NCT01120041

## Background

Dental health is an intergenerational problem in high-risk communities. Low-income women who report being in good dental health, and who believe in the benefit of dental care for their children, are more likely to have a usual source of dental care for themselves than are women who report both poor oral health and more negative attitudes toward pediatric dental care [1]. Similarly, preschool children whose mothers had a regular source of dental care were more likely to receive dental care [2,3]. Many women, however, do not seek oral care during pregnancy; while others who do, experience barriers including lack of dental coverage and limited access [4]. Dental providers’ lack of knowledge about the safety of dental care during pregnancy also limits pregnant women’s access to care [5]. Women without insurance coverage are least likely to receive dental services [6-9]. Continuity of care, a term to describe a usual source of care where children receive preventive services over time, has been found to be associated with improved overall utilization of medical services, better health outcomes, and increased use of preventive care [10-14]. There is modest but still positive evidence for the benefits of continuity of dental care [3,15].

One-to-one teaching of parents about the importance of preventing tooth decay in children has minimally improved children’s oral health [16]. Indeed, many people--especially those who have not had regular positive contacts with dental health providers--have the common-sense belief that when their teeth do not hurt them, there are no problems that require attention [17,18]. Two studies [19-21] have used brief Motivational interviewing (MI) to teach mothers about the importance of oral health of children and to assist them in overcoming obstacles to dental services for their children. In a study by Weinstein and colleagues [19,20], investigators studied 240 high-risk infants aged 6 to 18 months and their parents. Parents were randomly assigned to MI provided by a trained local woman or traditional health education. The MI intervention took 45 minutes and included six follow-up phone calls and two postcards over a year. It focused on home oral hygiene and dietary habits. The health education consisted of providing a pamphlet on preventing tooth decay and showing an educational video. There was a 46% reduction in the incidence of caries in the MI group compared to the health education group after two years. In similar study by Ismail and colleagues [20], parents who received a MI intervention reported changed behaviors but the study found no difference in the primary dental caries outcome. A critical review suggests that the interventions in the later study lacked fidelity to the intended treatment model [22].

During the same period as the MI research described above, the Klamath County, Oregon (USA) Department of Public Health conducted a community-based intervention program to promote dental visits by establishing a dental home for pregnant women covered by Medicaid (Oregon Health Plan), the state- administered national program of dental health insurance for qualified, low-income individuals [23]. Women served by Medicaid in Oregon State are eligible for comprehensive dental care during their pregnancy and for two months postpartum. Pregnant women in the county program received home visits or a one-on-one session at the Women, Infants, and Child Center at the health department by a dental hygienist and were assigned a dental home under an Oregon Health Plan managed care program. Le and colleagues used structured telephone interviews to identify factors that influenced women in the program to use, or not to use, available dental services [24]. The interviews asked specifically about stress and dental-related issues within a Stages of Change conceptual model [25] to determine factors that prevented or encouraged movement toward the action of going to the dentist. Overall, 55.8% of eligible women received dental care compared to less than 9% of pregnant women statewide and stress and dental issues were related to utilization. Follow-up showed 85% of the infants of the mothers who used care were caries free at 2 years old. In a similar cohort drawn from neighboring counties that did not participate in the program, only 58.9% of 2-year olds were found to be caries free [26].

The evaluation of the Klamath County Oregon program and the two earlier trials of MI counseling about Early Childhood Caries raised unanswered questions about whether MI is more effective than traditional health education in promoting dental visits and, if MI is superior to health education, at what stage of pregnancy or early childhood would it be most effective. This study should help fill these gaps.

## Methods/design

Baby Smiles is a five-year, four-site randomized intervention trial with a 2 × 2 factorial design in which participants were randomly assigned to one of four treatment arms: brief Motivational Interviewing (MI) or health education (HE) during pregnancy (prenatal) or postpartum. The National Institute of Dental and Craniofacial Research has sponsored the trial. The trial’s two primary objectives are to increase utilization of dental care by low-income women during pregnancy and up to two months postpartum and increase the use of preventive dental care by their young children by 18 months of age. The study rationale is that dental treatment during pregnancy, and preventive visits during early childhood, contribute to improved oral health during pregnancy and lower incidence of Early Childhood Caries [27]. The trial takes place in four rural counties in Oregon State (Douglas, Lincoln, Jefferson, and Josephine) USA and the interventions were delivered in the Women, Infants, and Children Center (WIC) or county public health department. WIC is a federal government program to ensure proper nutrition for poor mothers and their children. We chose to base the trial in public health settings because the agencies and locations are familiar to pregnant women. Also, this choice of setting allowed us to reach low-income women who are unlikely to have a usual source of dental care.

**Figure 1:**
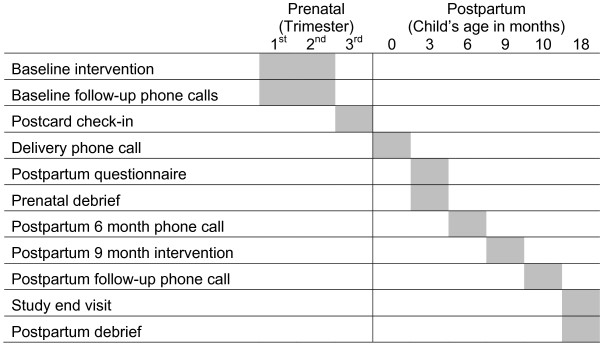
Overview of participant flow through the Baby Smiles study.

The Baby Smiles study advisory team consisted of a collaborative of county public health departments, community task forces, and dental care organizations that worked with the investigators to develop and conduct the study. The Data Coordinating Center is at the Northwest Center to Reduce Oral Health Disparities at the University of Washington in Seattle, Washington. The Institutional Review Board of the University of Washington and Public Health Institutional Review Board of Oregon state approved the study. An overview of participant flow through the study is shown in Figure 1.

### Study population

To be eligible, the woman had to be at least 15 years old, in her first or second trimester of pregnancy, be eligible for coverage by Oregon Health Plan Plus (the state administered Medicaid program which provides medical and dental services to adults and children), and speak English.

### Study treatments

The interventions utilize either MI or HE. We chose HE as the active control intervention because we did not feel it was ethical to have an untreated group and such an approach was unacceptable to the communities. Five college-educated social or health service professionals were chosen by the county health departments to be trained and deliver both interventions. One county required two counselors because there were two different enrollment locations. The counselors were employees of the county health departments. In the MI condition, counselors utilize standard MI techniques including open-ended questions, reflective listening, and affirmations [24]. In the HE condition, the counselors provide written and visual information about oral health but do not engage in active problem solving with the woman. The HE condition uses educational materials from the National Maternal Child Oral Health Resource Center at Georgetown University (http://www.mchoralhealth.org) and a locally-prepared slide presentation (prenatal) and video (postpartum). All participants receive information about using their dental care coverage, guidelines to being a successful dental patient, and tips for good oral health. Both the MI and HE interventions were scripted to assure fidelity among the counselors who were from varied backgrounds and expertise [22]. All the materials and the full study protocol are available on the website of the Northwest Center to Reduce Oral Health Disparities (URL: http://depts.washington.edu/nacrohd/babysmiles).

#### MI: prenatal

Participants assigned to this condition receive one-to-one in-person counseling during pregnancy. The counselor attempts to establish a therapeutic alliance, identify and reinforce the women’s dental needs, their dental risks, and identify and help navigate barriers to care. During the first in-person MI session, the counselor utilizes both a written and computer-driven protocol to deliver the intervention. The protocol includes showing a maximum of five very brief (1 to 2 minutes) videos to reinforce key points (*e*.*g*., “Baby teeth are important because if there is an infection in the baby teeth, there will be an infection in the permanent teeth…”) to assure fidelity to the MI protocol. Within six weeks of this in-person session, the counselor makes two follow-up telephone calls to provide support, to identify problems, and problem solve. Additionally, participants receive a phone call one month prior to the expected date of birth. Its purpose is to inquire about the pregnancy and continue discussing the woman’s dental concerns.

#### MI: postpartum

The first postpartum session occurs at 9 months postpartum and follows the same approach as the prenatal MI intervention but with a focus on the child. An Early Childhood Caries prevention menu is used information about oral hygiene and dietary practices and the age one dental visit. Mothers are asked to identify menu items that interest them and barriers to implementing these items are identified and discussed. This session is followed by one telephone call about six weeks afterwards to identify problems with the achievement of the mother’s stated goals.

#### HE: prenatal

Participants assigned to the prenatal HE condition receive a health education intervention. The materials include a 15-minute video created for the study and the pamphlet “Two Healthy Smiles” available from the National Maternal Child Oral Health Resource Center at Georgetown University (URL: http://depts.washington.edu/nacrohd/babysmiles). The in-person HE session is followed by up to two phone calls within six weeks to assess whether or not the participant went to the dentist, and offer assistance with scheduling if needed.

#### HE: postpartum

In the postpartum HE condition, a ten minute video is shown on preventing Early Childhood Caries and mothers are given two National Maternal Child Oral Health Resource Center pamphlets: “Your Young Child” and “Topical Fluoride Recommendations For High Risk Children.” (URL: http://depts.washington.edu/nacrohd/babysmiles). Within six weeks of the HE postpartum visit, the counselor called the participant, asked about whether or not the participant’s child went to the dentist, and offered to help get an appointment scheduled if necessary.

### Study schedule

Figure 1 summarizes the flow of study procedures from a participant’s perspective. The prenatal intervention in-person session was delivered at baseline when the participant was recruited, and the postpartum intervention was delivered, in person, when the participant’s child was nine months old. Counselors debriefed about the prenatal intervention when the participant’s child was three months old. The postpartum debrief was conducted at the study end visit.

### Recruitment and enrollment

Recruitment began May 1, 2010 and ended August 2, 2011. The primary recruitment methods were written materials, posted flyers and leave-behind flyers in the four intervention counties’ health departments, WIC settings, at prenatal care providers in the community, and at other community agencies whose clientele are pregnant women. Recruitment was also done in-person by WIC staff members. Each county’s public health department has employed a counselor (referred to as a County Counselor) to enroll study subjects and to perform the study procedures.

### Randomization and treatment contacts

Participants were randomly assigned to one of the four intervention groups using computer-generated permuted blocks of varying block sizes to ensure that the study groups would be proportionally balanced across study period and within each county and counselor. The randomization procedure was stratified on county and counselor. Study group proportions are listed in Table 1. We chose an unequal allocation to the four intervention groups, to end up with more participants with experience in the postpartum MI condition, in order to have adequate power to a test hypothesis about the combination of prenatal *and* postpartum MI versus postpartum-only MI. Overall, statistical power is adequate to detect MI versus HE differences in the primary outcomes. After participants gave consent and were enrolled, the County Counselor told the participant her prenatal study group assignment. The postpartum study group assignment was given at the 9-month postpartum visit.

**Table 1:** Four-group design for the Baby Smiles study

	**Child (Postpartum) MI**		
**Pregnancy**		**Yes**	**No**
**(Prenatal) MI**		Group 1	Group 2
	**Yes**	Prenatal MI-Postpartum MI	Prenatal MI–Postpartum HE
		N = 148	N = 52
		Group 3	Group 4
	**No**	Prenatal HE–Postpartum MI	Prenatal HE–Postpartum HE
		N = 148	N = 52

*Abbreviation*: *MI,* Motivational Interviewing; *HE,* Health Education.

### Study outcomes

#### Primary study outcome variable

The primary outcome measures for the study will be dental utilization during the prenatal period and up to two months postpartum for the mother, and preventive dental utilization by 18 months for the child. These data are part of the Medicaid claims database and will be obtained from the Oregon Division of Medical Assistance Programs, which is the section of the Department of Health and Human Services that administers the Oregon Medicaid claims database. The data will include all claims related to dental procedures covered under the Oregon Health Plan by Current Dental Terminology (CDT) codes. For example the OHP-defined category “Preventive Dental Services” (CDT codes - 100 s, 1000 s) includes routine and problem-based dental exams, cleanings, and fluoride treatments.

#### Secondary outcome variables

The two secondary outcome measures for the study will be 1) number of preventive home oral health practices taken by mothers to prevent caries in their young children, and 2) the mother’s readiness to change. The first outcome will be assessed by a questionnaire completed by the mother, or with assistance from the County Counselor [28,29]; the second outcome, readiness to change, will be assessed using a maternal-report “Readiness Ladder” modified for this study [30].

Additionally, several variables will be tested as mediators or moderators of the primary outcomes. These, also collected by the questionnaires, include prenatal depression [31], perceived stress [32,33], oral health impact [34], and dental anxiety [35]; and postpartum: dental anxiety [35], self-efficacy, oral health knowledge, fatalism [28,36], and child oral health impact [37].

### Intervention fidelity monitoring

Fidelity monitoring follows the framework developed by Belig and colleagues [38]. The approach focuses on three areas: (1) study design, (2) training interventionists, and (3) delivery, receipt and enactment of treatment skills during the intervention. We will use a modified Yale Adherence and Competence Scale (YACS) to rate adherence and competence in providing behavioral treatments for substance use disorders. YACS scales are reliable and have been shown to have construct and discriminant validity [39]. Intervention fidelity was monitored by coding 20% of the sessions. Other details of fidelity monitoring have been described elsewhere [22].

### Sample size

The sample size is 400 women with 80 to 120 women enrolled within any one county.

### Analysis

Three *a priori* group contrasts will be used to test the primary hypotheses regarding intervention effects on the use of dental services (see Table 1 for a summary of the 4 study groups):

(1) Groups 1 and 2 (MI Prenatal) versus Groups 3 and 4 (HE Prenatal) will test the hypothesis that the MI intervention results in a greater frequency of utilization of dental care by the woman during pregnancy.

(2) Groups 1 and 3 (MI Postpartum) versus Groups 2 and 4 (HE Postpartum) tests the hypothesis that the MI intervention results in a greater frequency of utilization of preventive dental care by the children during the first 18 months of life.

(3) Group 1 (MI Prenatal and Postpartum) versus Group 3 (MI Postpartum only) will test the hypothesis combination of MI delivered during pregnancy and postpartum will result in a greater frequency of preventive dental care for children during the first 18 months of life.

A significance level of 0.016 was used in the sample size and power calculations for the primary hypotheses to take into account the three comparisons to be performed. The study will also provide some evidence for the effect of MI during pregnancy, without MI postpartum, on child utilization. However, the study is not powered to demonstrate this effect, which will likely be modest in the absence of intervention postpartum.

### Missing data

Based on efforts to reduce attrition and missing data shown to be effective in our previous intervention work [20], and employed in this study, we expect to have an 80% retention rate of participation over the course of the study period. We will have even less missing data regarding dental utilization because we are able to obtain utilization data from the Medicaid database on all subjects, except those who explicitly refuse further participation in the study. In the case of non-ignorable non-response, a method based on augmented inverse probability of censoring weighted estimating equations will be used to perform a sensitivity analysis that examines how the estimated intervention effect on the primary outcome measure changes over a range of plausible values for the non-response mechanism [40].

### Primary outcome analysis

The analysis strategy for the primary hypotheses will involve intent-to-treat analyses, where women and their children will be compared according to their randomly- assigned intervention group regardless of whether, or how much of the intervention they actually received. The only exception to the intent-to-treat rule is in our analysis for child utilization of preventive dental care; this will be limited to only live-born children (*e*.*g*., miscarriages and stillborn will be excluded). Information about fetal loss will be collected by telephone interviews by the counselors with the participants because information about miscarriages is not on the state official birth record.

Intervention effects will be tested using the three *a priori* group contrasts described previously. Separate logistic regression analyses will be used to test each contrast using a significance level of 0.016.

A preliminary step in the analysis will be to check if the women in the four intervention groups are comparable on baseline values of prior dental utilization and important predictors of dental utilization (*e*.*g*. Readiness Ladder status, oral health quality of life). If an imbalance is found between the groups, additional covariates will be included in the logistic regression analyses to adjust for baseline differences. The primary independent variable in the regression analyses will be an indicator variable for intervention group. In addition, indicator variables for county and counselor will be included in all regression models to account for the stratification of the randomization by county and counselor.

Descriptive summaries will be produced for all the primary outcomes (mother and child dental utilization) by intervention group averaged over the four study counties, as well as by county, to assess the similarity of the observed intervention effects across the different counties. Formal testing for differences in the intervention effect among counties is possible by testing for interactions between county and intervention group in the regression analyses described below. However, we do not expect the intervention effects to differ vastly among the counties, and the sample size has not been selected to test for such county differences.

The logistic regression models testing the primary hypotheses will be similar. They will differ in terms of the study groups to be compared and the corresponding dependent variable (i.e., mother’s or child’s receipt of dental services). In each model, the dependent variable is utilization of dental services and the primary independent variable is the study group. In addition, indicator variables for county and counselor will be included in the logistic regression to account for the stratified randomization by county and counselor. Additional analyses will also compare the intervention groups for other types (non-preventive) of dental care utilization.

#### Secondary aims

Data from the baseline questionnaire during pregnancy and the questionnaires at 3, 9 and 18 months postpartum will be used to describe and assess how the mother’s readiness to change and other factors, including depression, oral health knowledge, oral health quality of life, oral hygiene and dietary practices, and dental anxiety, a well as changes in these variables over the study period, mediate or moderate the effect of the MI. For example, it is hypothesized that MI, as compared to the HE materials, will result in a greater number of home oral health practices adopted by the mother to prevent dental caries in her young children during the first 18 months of life. To test this, the total number of home oral health practices by the mother as reported on the questionnaire at 18 months postpartum will be compared between mothers in the postpartum MI intervention (Groups 1 & 3) and mothers in the postpartum HE intervention (Groups 2 & 4) using linear regression analysis. Similarly, it is hypothesized that MI as compared to the HE will result in a greater percentage of mothers classified by the Readiness Ladder as in the action stage (versus pre-contemplation and contemplation stages), a greater increase in oral health knowledge and self-efficacy, and lower level of oral health fatalism. Logistic and linear regression analysis, similar to the analytical approach used to test the primary hypotheses, will be used to test the secondary hypotheses.

Additionally, it is hypothesized that outcomes will be mediated by a women’s readiness to change. We will test whether changes in readiness to change over the course of the intervention mediates the effects of MI on the outcome measures for the mother at three months post-partum and for the child by 18 months of age. First, the analyses for the primary hypotheses will be used to examine whether there is an effect of the MI intervention on mother’s outcomes at three months post-partum and child outcomes by 18 months of age. Second, to demonstrate the association between the intervention and readiness to change, we will construct regression models with readiness to change as the dependent variable (e.g., change between baseline and three months post-partum for the mother’s outcomes, and change between and/or three months postpartum and 9 or 18 postpartum for the child’s outcome) and intervention and the baseline value of readiness to change as independent variables. Third, to demonstrate the association between readiness to change and the outcome after adjusting for the intervention and to demonstrate the reduction of the intervention effect on the outcome after adjusting for readiness to change, we will construct regression models with both treatment and readiness to change as independent variables and the outcome measure as the dependent variable.

To formally test the mediation effect as described in the third step above, we will use a version of the Sobel test [41], which tests whether the indirect effect of intervention on the outcome through the mediator (defined as the product of the intervention to mediator path and the mediator to outcome path) is significantly different from zero. The mediation effect is referred to as the indirect effect of intervention because it reflects the intervention effect on the outcome through the mediating variable [42]. We will use the bootstrap method of Preacher and Hayes [43] to estimate the indirect effect and bias-corrected 95% confidence interval (CI) for each individual mediator and for all the mediators as a group, based on 1,000 bootstrap samples using a Statistical Package for the Social Sciences (SPSS®; SPSS Inc., Chicago, Illinois) macro (http://www.afhayes.com/spss-sas-and-mplus-macros-and-code.html).

Moderators of intervention outcomes are typically baseline characteristics that interact with the intervention to affect outcomes. Logistic and linear regression analyses will be performed to identify if baseline variables, such as depression, oral health knowledge, and dental anxiety, health fatalism, predict mother or child outcomes, and whether any of these variables moderate the intervention effect. The independent variable in these analyses will include an indicator for the intervention group contrast of interest, the baseline variable, and an interaction between the baseline variable and intervention group.

## Discussion

The prevalence of Early Childhood Caries among disadvantaged North American populations ranges from 17-61% [44-47] and is as high as 87% among preschoolers living in rural communities [48-51]. Marked regional disparities in the prevalence of Early Childhood Caries have been documented with up to 73% of children in rural regions experiencing dental caries, compared with 51% in urban areas [52]. Early Childhood Caries has major impacts on quality of life, causing pain, eating and sleeping problems [53-56]. Understanding how health disparities are created, exacerbated or mitigated, and reproduced across generations is essential to eliminating differences [57].

Clinicians’ attempts to educate parents about the importance of children’s oral health have had mixed success and the two studies using Motivational Interviewing [16,20,21] obtained different results. The results of this trial should provide guidance for implementing brief cognitive interventions that may be more effective. In designing this trial, we purposely chose a community setting. We did this to increase buy-in and increase generalizability. We also used a carefully-scripted approach to both the MI and HE interventions. We did this because the interventionists had little or no previous training or experience with oral health of pregnant women or young children.

This trial is unique in that the primary outcome is drawn from Medicaid insurance claims rather than self-report or a clinical examination. The choice to utilize administrative data should minimize missing outcome data due to the loss to follow-up that often plagues longitudinal studies in low-income populations.

## Abbreviations

MI: Motivational interviewing; HE: Health education.

## Competing interests

The authors declare they have no competing interests.

## Authors’ contributions

PM helped conceive study and was the primary author of this paper. CAR was an author of the formal study protocol and contributed to the paper. PW was a co-author of the formal study protocol and contributed to the paper. LAM is the study biostatistician and author of the statistical methods. GG is the study manager for the trial and contributed to the paper. CEH was a co-author of the formal study protocol and contributed to the paper. DS helped organize the community aspects of the trial design and contributed to the manuscript. MS coordinated work with the participating counties and contributed to the paper. All of the authors reviewed and approved the final version of the paper.

## Pre-publication history

The pre-publication history for this paper can be accessed here:

http://www.biomedcentral.com/1472-6831/13/38/prepub
